# Evaluation of a Silicon ^90^Sr Betavoltaic Power Source

**DOI:** 10.1038/srep38182

**Published:** 2016-12-01

**Authors:** Jefferson Dixon, Aravindh Rajan, Steven Bohlemann, Dusan Coso, Ajay D. Upadhyaya, Ajeet Rohatgi, Steven Chu, Arun Majumdar, Shannon Yee

**Affiliations:** 1 G. W. W. School of Mechanical Engineering, Georgia Institute of Technology, Atlanta, Georgia, 30332, USA; 2 Department of Mechanical Engineering, Stanford University, California 94305, USA; 3 School of Electrical and Computer Engineering, Georgia Institute of Technology, Atlanta, Georgia 30332, USA; 4 Department of Physics, Stanford University, California 94305, USA

## Abstract

Betavoltaic energy converters (*i.e*., β-batteries) are attractive power sources because of their potential for high energy densities (>200 MWh/kg) and long duration continuous discharge (>1 year). However, conversion efficiencies have been historically low (<3%). High efficiency devices can be achieved by matching β-radiation transport length scales with the device physics length scales. In this work, the efficiency of c-Si devices using high-energy (>1 MeV) electrons emitted from ^90^Sr as a power source is investigated. We propose a design for a >10% efficient betavoltaic device, which generates 1 W of power. A Varian Clinac iX is used to simulate the high-energy electrons emitted from ^90^Sr, and a high efficiency c-Si photovoltaic cell is used as the converter. The measured conversion efficiency is 16%. This relatively high value is attributed to proper length scale matching and the generation of secondary electrons in c-Si by the primary β-particles.

The concept of a nuclear battery has attracted researchers since the 1950s because of the potentially long duration of continuous discharge^1^. The most widely used technologies have been the radioisotope thermoelectric generator (RTG) and direct ionizing radiation converters, such as alphavoltaics and betavoltaics^2^. RTGs in particular have garnered interest because of their high power output (>10 W); the Multi-Mission RTG that helps power a Mars rover generates 123 W for a period of 14 years^3^. However, RTGs are too large and heavy for some extraterrestrial applications and do not compliment the ever-decreasing size of some satellites (*e.g.*, cube satellites)^4^. There is now a mission need to revisit nuclear battery concepts with a target power generation of ~1 W.

As the names imply, alphavoltaics and betavoltaics rely on α and β-particles generated by their respective source materials to produce electric power through the direct conversion of the particles’ kinetic energy. In general, α-particles are emitted at greater energies than β-particles and therefore, alphavoltaics have the potential for higher power densities. However, collisions with the massive α-particles often cause rapid degradation to the alphavoltaic device, whereas damage from low mass β-particles causes over two orders of magnitude less damage to the betavoltaic device^5^,^6^,^7^. Furthermore, the commercial viability of alphavoltaics is reduced by the imposition of strict regulations on α-emitters (*e.g.*, special nuclear materials). In contrast, β-emitters are more distributable. In 2011, City Labs, Inc. was given the first general license to manufacture and distribute tritium betavoltaics operating in the nanowatt range for low power applications^8^. Currently, all commercial and laboratorial betavoltaics operate at efficiencies less than 3%^2^,^9^. Herein, we revisit the mechanisms involved in betavoltaic power generation and consider ways to improve the efficiency to levels exceeding 10%. We then discuss how this can be achieved in a device, and experimentally demonstrate high efficiency conversion using a simulated β-particle emitting source.

## Results

### Design of optimally length scale matched betavoltaic device

The operation of a betavoltaic cell is analogous to that of a photovoltaic cell with a few exceptions. β-particles penetrate deeper into a converter and lose energy primarily via Coulomb scattering/collisions and Bremsstrahlung emissions^10^. The former mechanism generates secondary electrons and the associated holes directly. Subsequent collisions involving the secondary electrons can generate tertiary electron-hole pairs, and so-forth. In this work, we do not distinguish between the secondary, tertiary, quaternary, *etc.* electrons; we refer to all of them as secondary electrons for convenience. If the electron-hole pair is generated within a diffusible distance to the depletion region of the diode, the native electrostatic potential can separate the electron-hole pair before it recombines^11^. Electron-hole pairs that are not generated close to the depletion region undergo recombination. However, unlike a photovoltaic cell where a photon causes an electronic iso-momentum transition from the valence band to the conduction band, high-energy β-particles (or secondary electrons) have sufficient momentum for non-iso-momentum transitions, thus affording more conduction-valence band transitions. Akin to tuning the thickness of the semiconductor material in photovoltaics to increase electron-hole pair collection, one must also tune the device length scale to match the β-radiation penetration of the source to achieve high conversion efficiencies. This is referred to as length scale matching^2^, (*i.e.*, the range of the β-particles in the semiconductor material must match the length scale of the device) and it is the foremost principle that guides this work.

To evaluate an appropriate source material, we considered over 400 isotopes based on their maximum β-particle energies, half-lives, maximum specific power (corresponding to maximum energy of β-particles), decay products/daughters, and availability. Table 1 shows the subset of what we think are the three most promising isotopes (^90^Sr, ^3^H, and ^63^Ni) for a high efficiency betavoltaic^2^,^12^,^13^.

**Figure 1:**
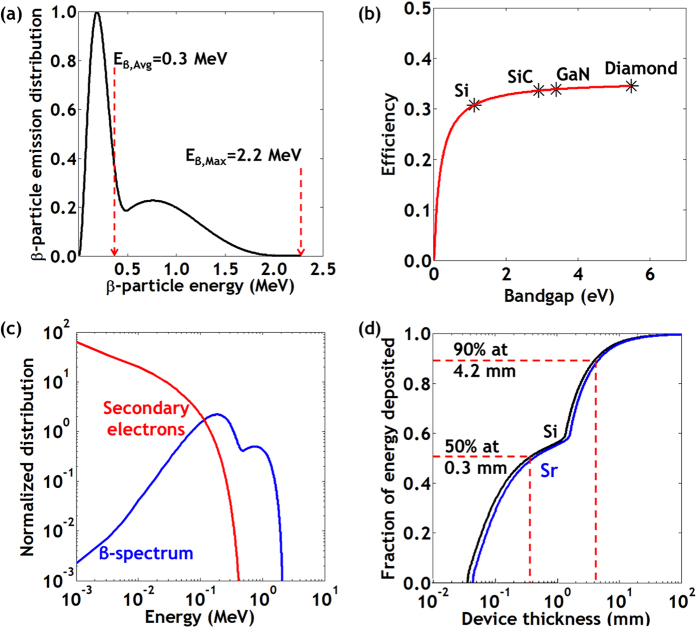
Length scale matching of a Silicon ^90^Sr betavoltaic device. (**a**) Normalized energy spectrum of β-particles emitted by the serial decay of ^90^Sr and ^90^Y. (**b**) Theoretical efficiency of energy collection in semi-infinite materials. (**c**) Normalized energy spectrum of secondary electrons generated by the β-spectrum of ^90^Sr + ^90^Y in comparison to the normalized β-spectrum itself. (**d**) β-particle energy deposition in Si and Sr. The “kink” near 1.5 mm is due to the higher energy β-particles from the ^90^Y decay. 50% of the β-particle energy is intercepted by a device that is 0.3 mm thick, which is comparable to the thickness of commercial photovoltaic cells.

We chose to further study ^90^Sr as the source material on the basis of its availability, decay mechanism, and spectral emission energies. ^90^Sr is produced as a fission product in nuclear reactors and accumulates in appreciable quantities in nuclear fuels^14^. ^90^Sr isotopically concentrates because it resides near a peak in the fission yield curve and does not require subsequent isotopic purification upon extraction. Upon β-decay, ^90^Sr decays into ^90^Y, which in turn undergoes β-decay to produce stable ^90^Zr. The complete β-decay of ^90^Sr and ^90^Y is given as,


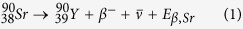



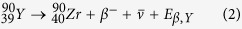


where *E*_*β,Sr*_ and *E*_*β,Y*_ denote the β-particle’s share of the decay energy that is continuously distributed between the β-particle (*β*^*−*^) and the anti-neutrino 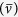. ^90^Sr and ^90^Y are in secular equilibrium as the half-life of ^90^Y is much shorter than that of ^90^Sr. Rarely, (0.01% of the time) an internal transition occurs within ^90^Y that emits a gamma ray before the emission of the β-particle. Hence, effectively two β-particles are generated with each ^90^Sr β-decay. Therefore, we can consider the combined emitted β-particle energy spectra for the purpose of further analysis. The number of β-particles emitted with kinetic energy *E*_*β*_ is given by the following expression^15^,


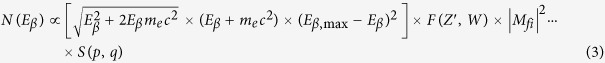


where *m*_*e*_ is the rest mass of the electron, *c* is the speed of light in vacuum, and *E*_*β,max*_ is the maximum decay energy (or end-point energy). *F(Z′, W)* is the Fermi function that accounts for relativistic coulomb effects, *M*_*fi*_ is the nuclear matrix element that accounts for the initial and final states, and *S(p, q)* is the shape factor which accounts for the first forbidden transition of both ^90^Sr and ^90^Y (see Supplementary Information, section 1 for more details). Using the simplified Fermi function as described by Venkataramaiah *et al*.^16^, we calculated the cumulative spectrum of ^90^Sr and ^90^Y which is illustrated in Fig. 1(a). The maximum energy of the β-particle is ~2.2 MeV, which corresponds to the end-point energy of the β-decay of ^90^Y; the average energy of the β-particle is ~0.3 MeV. The emission probability distribution is normalized to two, representative of the two β-particles emitted in the ^90^Sr + ^90^Y secular decay. Our pure analytical spectrum is validated by experimental spectroscopy of β-emission from ^90^Sr, with the differences particularly at lower energies due to spectroscopic instrument response functions^17^,^18^. The exact spectrum is crucial to predicting the energy deposition in the diode and minimizing the self-absorption of β-particles that may arise in the source material.

To further justify the choice of ^90^Sr as the source material, we consider the interaction of β-particles with the semiconductor converter. Analogous to the Shockley-Queisser limit in photovoltaics, betavoltaics have a theoretical maximum efficiency contingent on the bandgap of the semiconductor; the conversion efficiency of a semi-infinite semiconductor material is limited to approximately^2^,^8^,^19^,


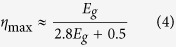


where *E*_*g*_ is the bandgap of the semiconductor material (see Supplementary Information, section 2 for more details on this calculation). The efficiency as a function of bandgap is given in Fig. 1(b) for the four semiconductor materials that we considered. As shown in the figure, the maximum efficiency does not vary significantly (<5%) for wide bandgap materials. In this work, we have chosen to use crystalline silicon as the converter material. c-Si is abundant, has a mature processing infrastructure, and high efficiency commercial photovoltaics made of c-Si were easily available for the latter part of our work.

To optimize energy collection, the c-Si device thickness should be matched to the β-radiation penetration length scale^2^. As the β-particles traverse through the ^90^Sr source material and the c-Si converter, they dissipate their energy via collisions and Bremsstrahlung emission. The stopping power associated with this energy dissipation is the sum of the stopping powers associated with collisions and Bremsstrahlung. Stopping power, in the context of radiation shielding, is usually truncated at the first order as part of the Constant Slowing Down Approximation (CSDA)^10^. However, to evaluate the region of maximum energy deposition in c-Si, the higher order terms of the expansion that account for the secondary electron generation must be considered (see Supplementary Information, section 3 for details about secondary electrons). This is corroborated in Fig. 1(c). The number of secondary electrons generated on the left side of the spectrum is orders of magnitude greater than the number of penetrating β-particles. Electrons with energies closer to the bandgap are more efficient at producing electron-hole pairs, and therefore, the secondary electron spectrum is crucial to evaluate energy deposition. Convolving the secondary electron spectrum with the emission spectrum, we calculated the amount of energy deposited in c-Si (black curve) and Sr (blue curve) by the ^90^Sr + ^90^Y β-spectrum as a function of material thickness as illustrated in Fig. 1(d), which is key to understanding length scale matching. The energy deposition in Sr (blue curve) represents the self-absorption of the β-particles by the source material. The ^90^Sr source material may at most be 45 μm before losses due to self-absorption begins, as indicated by the x-intercept. Furthermore, since Sr is a high-Z material, self-absorption results in the generation of Bremsstrahlung x-rays, which has caused past researchers to erroneously discount ^90^Sr as a viable betavoltaic source material (see Supplementary Information, section 3 for more information about Bremsstrahlung generation). These X-rays can be mitigated by using thin depositions, dispersions, or foils of ^90^Sr layered between thicker sheets of c-Si.

We observed from the above analysis that the radiation penetration length scales of the ^90^Sr + ^90^Y β-particles correspond well with the device length scales of existing high efficiency commercial c-Si photovoltaic devices (~40% energy collection efficiency for a ~150 μm device). Hence, we conjecture that a high efficiency solar cell, operating as a betavoltaic device, would likely convert high-energy (>1 MeV) electrons into electricity at a high efficiency (>10%).

**Figure 2:**
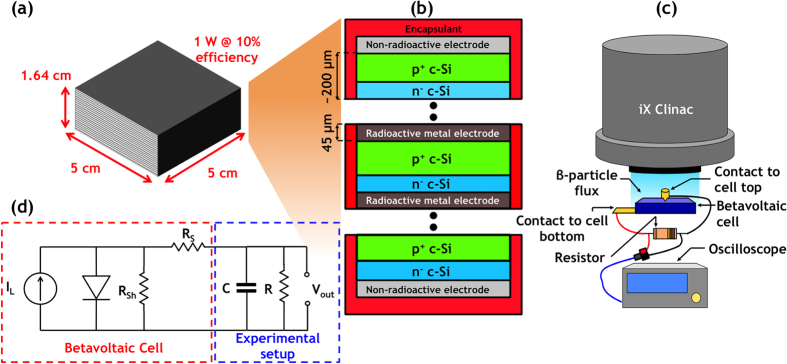
Envisioned 10% efficient betavoltaic device and setup of experiment. (**a**) Overall dimensions of a proposed 1 W device. (**b**) Cross section of the proposed device architecture. (**c**) Experimental setup used to characterize the efficiency of the betavoltaic cell. (**d**) Equivalent circuit of the setup in (**c**).

Figure 2(a) illustrates a potential c-Si betavoltaic device that could generate 1 W of power from ^90^Sr with a conversion efficiency of >10%. We envision the device to be modularly stacked with alternating layers of 45 μm thick ^90^Sr foil and a 265 μm thick p-n diode (Fig. 2(b)). For a device with a projected area of 25 cm^2^, 35 such modules would be necessary to generate 1 W at 10% efficiency. Overall, this would ensure >90% energy deposition within the entire stack; a particle that passes through the first p-n diode is more likely to be captured in an adjacent p-n diode. Individual devices could then be connected in series or parallel. A collection of such devices that generates 100 W would weigh less than 5 kg.

### Experimental demonstration of efficient power generation at high electron energy

To circumvent the radioactive challenges involved with procuring and handling ^90^Sr at a university, we simulated the β-spectrum of the ^90^Sr + ^90^Y source material using a Varian Clinac iX System Linear Accelerator, or Clinac, located at the Georgia Institute of Technology’s Radiological Science and Engineering Laboratory. For the converter, we used a conventional photovoltaic cell that was fabricated at the Georgia Institute of Technology’s University Center of Excellence for Photovoltaics (UCEP). The lowest measured efficiency was 16%.

## Methods

The Clinac was configured to emit a beam of 6 MeV electrons covering an area of ~25 cm^2^ at a dosage of 300 MU/min, which is equivalent to a flux of (1.068 ± 0.02) × 10^8^ electrons/cm^2^s. Although the Clinac does not replicate the β-spectrum of ^90^Sr + ^90^Y exactly, the large beam size and high energy electrons (~MeV) allow us to test our hypothesis that length scale matching in c-Si with an appropriate >1 MeV β-emitting source can demonstrate higher efficiencies than those previously reported. We attempted to shape the spectrum with a window to resemble the ^90^Sr + ^90^Y β-spectrum, but we were unsuccessful at maintaining adequate transmission intensity for our experiments (see Supplementary Information, section 6 for more details). The 6 MeV electrons have a smaller probability of being directly absorbed than the high-energy (>1 MeV) β-particles emitted from ^90^Sr + ^90^Y. However, at these high energies both 6 MeV electrons and high-energy (>1 MeV) β-particles emitted from ^90^Sr + ^90^Y have qualitatively similar scattering and potential for the generation of secondary electrons. While the 6 MeV monochromatic sources is not a direct spectral match to the ^90^Sr + ^90^Y spectrum, the production of secondary electrons from scattering is similar. Therefore, we expect the effects on radiation penetration depth and efficiency to be similar for the 6 MeV monochromatic source as with a true ^90^Sr + ^90^Y spectrum. Furthermore we note that our experiment is similar to that of Bao *et al*., who used an electron gun to simulate the β-spectrum of Pm-147. In their experiment however, most β-particles possessed energies in the order of keV^20^.

**Figure 3:**
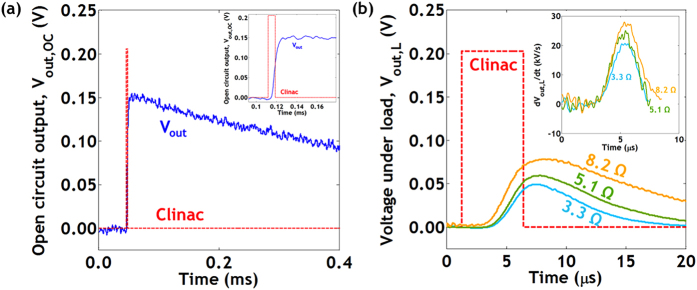
Open and closed circuit voltage response of betavoltaic device under an electron flux from the Clinac. (**a**) Clinac open circuit voltage response of the betavoltaic cell under electron illumination. Inset: A zoomed in plot of the same. V_out_ rises rapidly with the Clinac input and decays over time due to recombination. (**b**) Voltage across each resistor. Inset: Time derivative of the voltage response. The peak values of this plot can be multiplied with C to acquire the maximum current during each pulse. The red dotted line represents the Clinac trigger signal.

One practical challenge with using the Clinac is its characteristic pulsed emission of electrons. The Clinac delivers 5.6 μs long pulses of electrons at 5 ms intervals. However, it was discovered that these pulses occur at slightly irregular intervals varying as much as ~1 μs. This timing irregularity prevented us from preforming conventional triggered IV measurements to characterize the performance of the converter. Therefore, we performed a direct measure of the power generated by the converter and then dissipated in a load resistor connected in series with the converter under electron illumination (Fig. 3).

The photovoltaic cells we used were nearly identical to commercial cells manufactured by Suniva, which spun out of UCEP (see Supplementary Information, section 4 for the specifications of the photovoltaic cell). The cells were laser cut to a dimension that allowed for full area illumination under the electron source.

The electrical setup can be modeled as a photovoltaic/betavoltaic cell comprised of a current source *I*_*L*_, a diode, a shunt resistance *R*_*Sh*_, and a series resistance *R*_*S*_ connected in series to a load resistor *R* and a parallel capacitor *C*. The voltage across the resistor *V*_*out*_ is directly measured. The capacitor *C* results from the cables that connect the device-under-test in the Clinac to the control room where data is acquired. The cable capacitance was determined to be 1.07 ± 0.13 μF. Using three precision (<0.1%), low temperature coefficient (50 ppm/°C) load resistors (3.3 Ω, 5.1 Ω and 8.2 Ω), we directly measured the power generated by the betavoltaic cell.

We then measured the efficiency of the betavoltaic cell using a direct method; by taking the time integration of the power dissipated in the resistor, 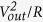, and source, *P*_*S*_.


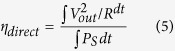


We calculated *η*_*direct*_ for resistors 3.3 Ω and 5.1 Ω to be 16 ± 2% and 19 ± 2%, respectively. We excluded the 8.2 Ω resistor data set from the *η*_*direct*_calculation because the *V*_*out*_ response was interrupted by the arrival of the subsequent Clinac pulse.

## Conclusions

These Clinac experiments demonstrate that modern c-Si photovoltaic devices have promise for being used as betavoltaic devices for the conversion of high-energy (>1 MeV) electrons. We attribute this to the coincidence that modern photovoltaic device sizes (~200 μm) match reasonably well with the radiation penetration depth (~40% at ~150 μm) of high-energy electrons (Fig. 1(d)). Additionally, the secondary electrons that are generated in c-Si contribute (Fig. 1(c)) to increased energy collection as these lower energy electrons also create electron-hole pairs. Keeping the ^90^Sr source material thickness to less than 45 μm (Fig. 1(d)) minimizes self-absorption. Minimal ^90^Sr thickness also has the added advantage of mitigating Bremsstrahlung emissions when combined with low-Z converters like c-Si. Overall, the prospects of integrating ^90^Sr as a source material in c-Si based betavoltaic devices is a promising route to achieve high-efficiency (>10%) at reasonable powers (~1 W) for long duration (>10 years) which could be quickly matured into field testable units in a proper facility (*e.g*., a National Laboratory).

## Additional Information

**How to cite this article**: Dixon, J. *et al*. Evaluation of a Silicon ^90^Sr Betavoltaic Power Source. *Sci. Rep.* **6**, 38182; doi: 10.1038/srep38182 (2016).

**Publisher's note:** Springer Nature remains neutral with regard to jurisdictional claims in published maps and institutional affiliations.

## Supplementary Material

Supplementary Information

## Figures and Tables

**Table 1:** Top contenders for the choice of β-emitting radioisotope for the betavoltaic cell.

Radioisotope	Maximum energy of β-particles (keV)	Half-life (years)	Maximum power density (W/g)	Availability
H-3	18.6	12.3	0.325	Medium
Ni-63	66.7	100.2	0.006	Low
Sr-90	545.9	28.8	0.164	High
Y-90	2,279.8	0.007	0.780	—
^90^Sr + ^90^Y	—	—	0.944	High
