# Supplementary material for: Evaluation of a Silicon 90Sr Betavoltaic Power Source
Source: Sci Rep. 2016 Dec 1;6:38182. doi: 10.1038/srep38182 (PMC5131278; doi:10.1038/srep38182)
Supplement: Supplementary Information [file srep38182-s1.pdf]

Evaluation of a Silicon ^{90}Sr Betavoltaic Power Source: Supplementary Information

Jefferson Dixon[1], Aravindh Rajan[1], Steven Bohlemann[1], Dusan Coso[2], Ajay D. Upadhyaya[3], Ajeet Rohatgi[3], Steven Chu[4], Arun Majumdar[2], Shannon Yee[1*]

[1]G. W. W School of Mechanical Engineering, Georgia Institute of Technology, Atlanta, Georgia, 30332, USA
[2]Department of Mechanical Engineering, Stanford University, California 94305, USA
[3]School of Electrical and Computer Engineering, Georgia Institute of Technology, Atlanta, Georgia 30332, USA
[4]Department of Physics, Stanford University, California 94305, USA
[*]e-mail: shannon.yee@me.gatech.edu

Section 1: Cumulative β-spectrum of ^{90}Sr and ^{90}Y

The number of β-particles emitted with kinetic energies between E_β and $E_\beta+dE_\beta$ is given by,[S1]

$$N(E_\beta) \propto \left[\sqrt{E_\beta^2 + 2E_\beta m_e c^2} \times \left(E_\beta + m_e c^2\right) \times \left(Q - E_\beta\right)^2\right] \times F(Z',W) \times \left|M_{fi}\right|^2 \times S(p,q) \qquad \text{(S1)}$$

where $m_e c^2$ is the rest mass energy of the electron with regard to the relativistic nature of the high energy β-emission, c is the speed of light in vacuum and $E_{\beta,max}$ is the difference between the final and initial mass energies (end-point energy). The Fermi function $F(Z',W)$, which accounts for the relativistic coulomb effects as the β-particle exits the envelope of the nucleus, is given by the following simplified expression as put forward by Venkataramaiah e*t al.*[S2]

$$F(Z',W) = [A + B/(W-1)]^{1/2} \qquad \text{(S2)}$$

In Eqn. S2, W is the total energy of the β-particle at a particular momentum (kinetic energy + rest mass energy), and A and B are constants corresponding to the atomic number of the daughter nucleus Z'. For ^{90}Y, A and B are 8.6994 and 4.7116 respectively. The same parameters are 9.3330 and 5.2025 respectively for ^{90}Zr. The nuclear matrix element $|M_{fi}|^2$ accounts for the transition between initial and final nuclear states. The nuclear matrix element is a result of differences in the nuclear wave functions and thus, differences in the comparative half-life values. The nuclear matrix element is given as,[S1]

$$\left|M_{fi}\right|^2 = 0.693 \frac{2\pi^3 \text{h}^7}{g^2 m_e^5 c^4 ft_{1/2}} \qquad \text{(S3)}$$

where g is the β-decay strength constant (g = 1.3192), m_e is the mass of the electron, c is the speed of light in vacuum, $\hbar$ is the reduced Planck constant (*i.e.,* h-bar), and $ft_{1/2}$ is the comparative half-life. The comparative half-life is a measure of the β-decay probabilities in

different nuclei.[S1] Finally, the shape factor *S(p,q)* is used to account for the first forbidden transition of both ^{90}Sr and ^{90}Y and is given as,

$$\left|M_{fi}\right|^2 = 0.693\frac{2\pi^3 h^7}{g^2 m_e^5 c^4 ft_{1/2}} \tag{S4}$$

Using the above terms, the spectrum for both ^{90}Sr and ^{90}Y can be calculated. Since the two parent-daughter radioisotopes are in secular equilibrium, the cumulative spectrum can be evaluated by simply adding the normalized individual spectra. This culminates in Fig. 1(a).

Section 2: Efficiency of energy collection of a semi-infinite semiconductor material

As the β-particles penetrate into the semi-infinite semiconductor material, the β-particles dissipate their energy by generating electron-hole pairs and Bremsstrahlung x-rays. Being a material with low-Z value, the amount of Bremsstrahlung produced in c-Si is small (~0.43%); this is further addressed in Section 3. Furthermore, on account of the semi-infinite material, the energy dissipation mechanisms are limited to electron-hole generation and energy transfer to phonons. Ignoring recombination losses, the efficiency of energy collection η is then given by,[S3]

$$\eta = \eta_{dp} \times \eta_d \times \eta_{pp} \times FF \tag{S5}$$

where η_{dp} is the ratio of the open circuit voltage to the bandgap, η_d represents the fraction of incident energy deposited in the depletion region, η_{pp} is the efficiency of electron-hole pair production, and FF is the fill factor of the semiconductor device. In the limit of a perfect semi-infinite device (i.e., FF=1, η_{dp}=1, η_d =1) a theoretical maximum efficiency of energy collection can be defined as,

$$\eta = \eta_{pp} \tag{S6}$$

Considering the cumulative number distribution of β-particles, $N_{cum}(E_\beta)$, emitted by ^{90}Sr + ^{90}Y, the total energy of the particles is given by,

$$E_{total} = \int_0^{E_{\beta,\max}} E_\beta \times N_{cum}(E_\beta) dE_\beta \tag{S7}$$

Among the β-particles, those with energies greater than the bandgap could contribute to electron-hole pair production. On average, it costs the particles the mean ionization energy to generate the pair.[4] For a material with bandgap E_g and mean ionization energy E_{ion}, the energy collected as electron-hole pairs from the incident β-particles is given as,

$$E_{collected} = \int_{E_g}^{E_{\beta,\max}} \frac{E_g}{E_{ion}} \times E_\beta \times N_{cum}(E_\beta) dE_\beta \quad \text{(S8)}$$

Eqn. (S8) can then be written as,[S4]

$$\eta = \frac{E_{collected}}{E_{total}} = \frac{E_g}{E_{ion}} \frac{\int_{E_g}^{E_{\beta,\max}} E_\beta \times N_{cum}(E_\beta) dE_\beta}{\int_0^{E_{\beta,\max}} E_\beta \times N_{cum}(E_\beta) dE_\beta} \approx \frac{E_g}{E_{ion}} \approx \frac{E_g}{2.8 \times E_g + 0.5} \quad \text{(S8)}$$

The approximation is valid because the minimum energy of the cumulative β-spectrum of ^{90}Sr and ^{90}Y is orders of magnitude larger than the bandgap. The mean ionization energy, as stated by Klein, is the sum of the energy contributed to generating the electron-hole pair ($1E_g$ eV), energy lost to acoustic phonons and optical phonons in the pair production process ($1.8E_g$ eV and 0.5 eV*,* respectively).[S4]

Section 3: Penetration depth, secondary electrons and Bremsstrahlung

Utilizing the energy spectrum, the penetration depth of the beta electrons can be calculated to determine the amount of energy deposited within the device as a function of device thickness. The first step in developing this relationship is to examine the interactions of an electron at a discrete energy as it travels through a material. The two primary modes of interaction are direct collisions and radiative emissions or Bremsstrahlung. The penetration depth is obtained in terms of the stopping power, *-dE/dx*, as given below[S5]

$$\left(-\frac{dE}{dx}\right)_{total} = \left(-\frac{dE}{dx}\right)_{col} + \left(-\frac{dE}{dx}\right)_{rad} \quad \text{(S9)}$$

which is a function of the collisional stopping power, $(-dE/dx)_{col}$, and the stopping power associated with radiative emissions, $(-dE/dx)_{rad}$. The Constant Slowing Down Approximation assumes that charged particles slow continuously with all its energy deposited locally along the particle paths.[S1] In this approximation and for low radiative yield (which is true for β-particles through c-Si), $(-dE/dx)_{rad}$ and $(-dE/dx)_{col}$ are respectively given as,[S5]

$$\left(-\frac{dE}{dx}\right)_{rad} \cong \frac{Z \times W_\beta}{800} \left(-dE/dx\right)_{col} \quad \text{(S9)}$$

$$\left(-\frac{dE}{dx}\right)_{col} = \frac{4\pi k_0^4 e^4 n_e}{m_e c^2 v^2} \left[\ln \frac{W_\beta \sqrt{W_\beta / m_e c^2 + 2}}{\sqrt{2} I} + F^-(v) \right] \quad \text{(S10)}$$

where Z is the atomic number of the material through which the β-particle is traversing, W_β is the total energy of the β-particle, I is the mean excitation energy for silicon, k_0 is the Coulomb constant, e is the charge of an electron, n_e is the number of electrons in the substrate per unit volume, m_e is the mass of an electron, c is the speed of light and v is the relativistic speed of the β-particle. $F^{-}(v)$ is the function,[S5]

$$F^{-}(v)=\frac{1-v^2}{2}\left[1+\frac{\left(W_\beta/m_e c^2\right)^2}{8}-(2W_\beta/m_e c^2+1)\times\ln 2\right] \tag{S11}$$

The CSDA approximation treats any energy lost by the β-particle via collisions to be infinitesimal compared to the energy of the β-particle.[S6] However, the CSDA approximation does not account for the generation of secondary electrons. In our case, the incident β-particles are several orders of magnitude more energetic than the bandgap and thus, secondary electrons are crucial to determining the penetration depth. The concept of secondary electrons relies on the high kinetic energies of the incident β-particles. Upon collisions with electrons, the β-particles are capable of imparting as much as half of their energy to the electrons (several orders of magnitude greater than the bandgap). These energetic electrons are now capable of generating their own electron-hole pairs or more energetic electrons. This type of collisions leads to a "particle shower". The probability of collisions $P_m(E, E_{loss})$ by an electron of energy E leading to an energy loss E_{loss}, is given by the Møller's relativistic equation, which also accounts for the generation of secondary electrons.[S6]

$$P_M(E,E_{loss})=\frac{2\pi e^6 n_e}{m_e^3 c^6 v^2}\left[E_{loss}^{-2}+\left(E-E_{loss}\right)^{-2}-\left(\frac{2+E^{-1}}{(E+1)^2}\right)\times\left(E_{loss}^{-1}+(E-E_{loss})^{-1}\right)+\left(E+1\right)^{-2}\right] \tag{S12}$$

As per the definition of a secondary electron, $E_{loss} \le E/2$. Both the previous terms are expressed in mc^2units. This expression is one of the main deviations from the CSDA.

We adopted Spencer and Fano's treatise[S6] on the energy spectrum of secondary electrons for an arbitrary β-particle (Fig. S1). We convolved this treatment with the β-spectrum of $^{90}Sr+^{90}Y$ which resulted in Fig. 1(d). The secondary electron spectrum also represents the average track length of the electron *i.e.*, the distance travelled by an electron as its energy varies from $E+dE$ to E. A probabilistic weighting of all the track lengths associated with the $^{90}Sr+^{90}Y$ β-spectrum allows us to determine the energy deposition in c-Si and ^{90}Sr, as illustrated in Fig. 1(b).

To calculate the amount of Bremsstrahlung generated, we used the penetration depth associated with radiation emission as given in Eqn. 11. The percentage of radiation yield Y, is given as,[S5]

$$Y\cong\frac{(6\times10^{-4}\times Z\times E_\beta)}{1+(6\times10^{-4}\times Z\times E_\beta)} \tag{S13}$$

where Z is the atomic number of the material through which the β-particle with kinetic energy E_β is traversing. Figure S2 shows that β-particles generated from the source material and penetrating into ^{90}Sr would generate Bremsstrahlung radiation worth 1.15% of the energy of the β-particles. However, through c-Si, the same value is only 0.43%. The remaining interactions are dominantly collisional, which result in the generation of lower energy secondary electrons and then ultimately, in electron-hole pair generation within the semiconductor.

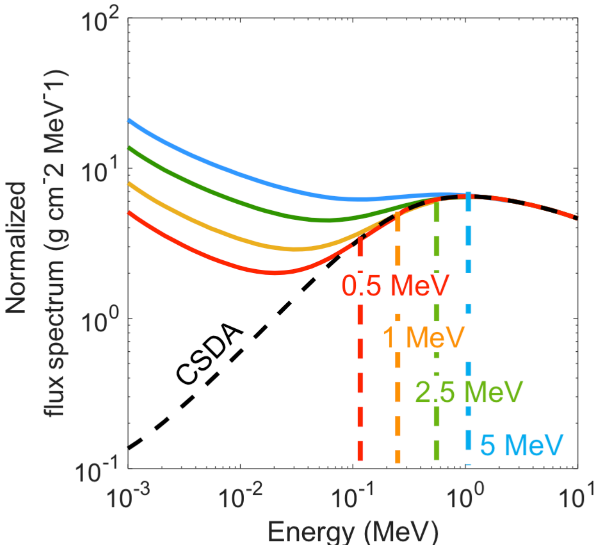

Figure S1. Normalized energy spectrum of electrons generated in c-Si for different incident β-particle energies. The area between a colored line and the black CSDA plot denotes the surplus secondary electrons generated in c-Si for the specified energy of incident β-particles. The y-axis, in addition to representing the average track length an electron travels as its energy decreases from $E+dE$ to E, also describes the spectrum of the electrons generated by a mono-energetic source of β-particles.

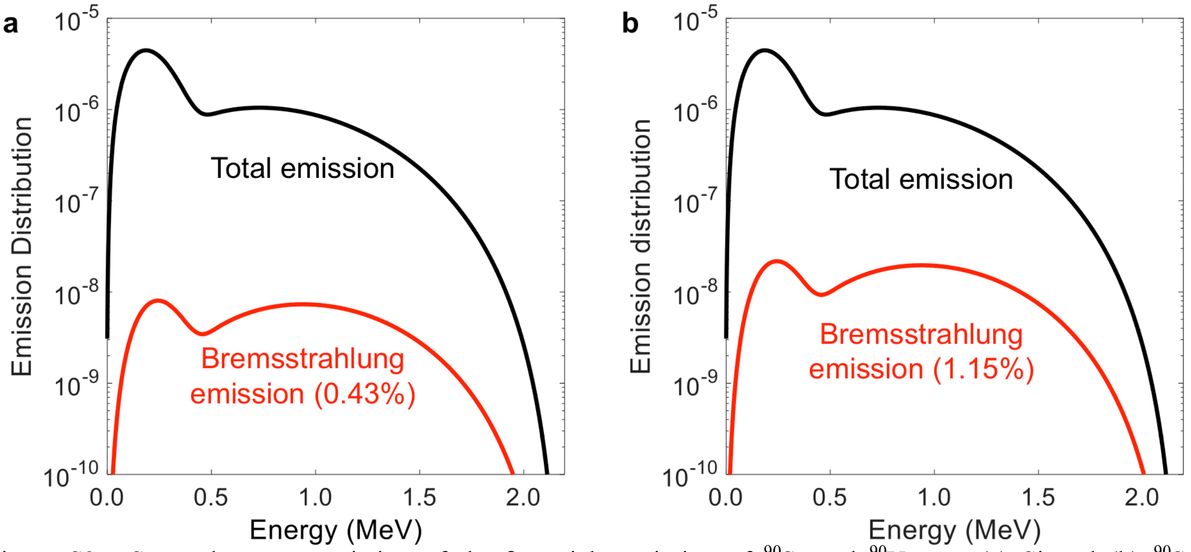

Figure S2. Spectral energy variation of the β-particle emission of ^{90}Sr and ^{90}Y onto (a) Si and (b) ^{90}Sr accompanied by the respective resultant Bremsstrahlung emission. The net Bremsstrahlung emission from Si and ^{90}Sr are only 0.43% and 1.15% of the total energy of the β-particles emitted, respectively. Si is a low-Z material and therefore produces less Bremsstrahlung compared to ^{90}Sr, which is a high-Z material.

Section 4: UCEP cell parameters

Table S1 below shows the specifications of the UCEP cells used in the experiment as determined from photovoltaic characterization.

TABLE S1. UCEP cell parameters

Efficiency	18.5 %
V_{OC}	642.1 mV
I_{SC}	962.58 mA
Fill Factor	74.9
R_{SH}	6 kΩ
R_S	1.1 Ω
Size	25 cm^2
Thickness of diode	180-200 µm

Section 5: Power input from the Clinac

TABLE. S2. Power input calculation from Clinac

Flux from Clinac (number of electrons)	1.608×10^8 e^-/cm^2s
Flux from Clinac (incident energy)	1.539×10^{-4} J/cm^2s
Area of cell illumination	25 cm^2
Power input, P_S	3.841 mW

Section 6: Energy degrader to simulate the β-spectrum of the radio-isotope

One drawback of our experiment is the inability to replicate the $^{90}Sr+^{90}Y$ β-spectrum. We envisioned a window made of lead, PMMA and aluminum (See Fig. S3) that acts as an energy degrader by converting the mono-energetic electron beam to an electron flux that is more representative of the β-spectrum of our source material. However, the window reduced the fluence of the Clinac to 0.36% of its original value and we failed to measure current across a load.

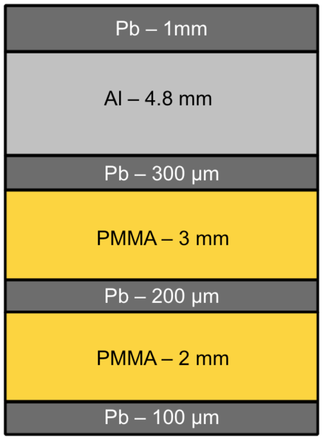

Figure S3. Cross-sectional view of the energy degrader window that was used to convert the mono-energetic beam of electrons to an electron flux that resembled the $^{90}Sr+^{90}Y$ β-spectrum. However, in addition to reshaping the spectrum, the window also reduced the fluence (number of particles per unit area and time) to 0.36% of the original value making it difficult to measure a current.

Supplemental References

S1 Krane, K. S. *Introductory nuclear physics*. (New York : Wiley, 1988).

S2 Venkataramaiah, P. Asimple relation for the fermi function. *Journal of Physics G: Nuclear Physics* **11**, 359-364, doi:10.1088/0305-4616/11/3/014 (1985).

S3 Prelas, M. A. *et al.* A review of nuclear batteries. *Progress in Nuclear Energy* **75**, 117-148, doi: 10.1016/j.pnucene.2014.04.007 (2014).

S4 Klein, C. A. Bandgap Dependence and Related Features of Radiation Ionization Energies in Semiconductors. *Journal of Applied Physics* **39** (1968).

S5 Turner, J. E. *Atoms, radiation, and radiation protection*. 2nd ed.. edn, (New York : J. Wiley, 1995).

S6 Spencer, L. V. & Fano, U. Energy Spectrum Resulting from Electron Slowing Down. *Physical Review* **93**, 1172-1181, doi:10.1103/PhysRev.93.1172 (1954).
